# Association Between SGLT2 Inhibitor Use and New-Onset Atrial Fibrillation Following Transcatheter Aortic Valve Implantation: A Doubly Robust Inverse Probability Weighted Analysis

**DOI:** 10.3390/jcm15124812

**Published:** 2026-06-21

**Authors:** Mustafa Ferhat Keten, Kadir Biyikli, Barkin Kultursay, Halit Eminoglu, Dogancan Ceneli, Nesri Danisman, Cagri Kafkas, Ismail Balaban

**Affiliations:** 1 Department of Cardiology, Hamidiye School of Medicine, Kosuyolu Heart, Education and Research Institute, University of Health Sciences, Istanbul 34925, Türkiye; kadirbiyikli93@hotmail.com (K.B.); eminogluhalit@gmail.com (H.E.); dogancanceneli@yahoo.com (D.C.); drnesridanisman@gmail.com (N.D.); ismailbalabanmd@gmail.com (I.B.); 2 Department of Cardiology, Tunceli State Hospital, Tunceli 62000, Türkiye; bkultursay@gmail.com; 3 Department of Cardiology, University of Marmara, Istanbul 34854, Türkiye; cgrkfks@gmail.com

**Keywords:** diabetes mellitus, inverse probability weighting, new-onset atrial fibrillation, SGLT2 inhibitors, transcatheter aortic valve implantation

## Abstract

**Background:** New-onset atrial fibrillation (NOAF) is a common complication after transcatheter aortic valve implantation (TAVI) and is associated with unfavorable clinical outcomes. Sodium–glucose cotransporter-2 (SGLT2) inhibitors may have antiarrhythmic effects, but their association with NOAF after TAVI remains uncertain. This study evaluated the relationship between SGLT2 inhibitor use and NOAF following TAVI. **Methods:** This retrospective observational study included 573 consecutive patients who underwent transfemoral TAVI between January 2020 and December 2025. Patients with prior atrial fibrillation or atrial flutter were excluded. NOAF was defined as any atrial fibrillation episode lasting ≥30 s during index hospitalization. A doubly robust inverse probability weighted logistic regression model was applied to reduce baseline imbalances and assess the association between SGLT2 inhibitor use and NOAF. **Results:** Overall, 169 patients received SGLT2 inhibitors, while 404 patients constituted the control group. NOAF occurred less frequently in the SGLT2 inhibitor group than in controls (11% vs. 19%, *p* = 0.041). In adjusted analysis, SGLT2 inhibitor use was independently associated with lower odds of NOAF (adjusted OR: 0.171, 95% CI: 0.076–0.381, *p* < 0.001). Older age and diabetes mellitus were associated with increased NOAF risk, whereas higher baseline left ventricular ejection fraction was associated with lower risk. Subgroup analysis indicated a possible interaction by diabetes status (P-interaction = 0.040), although this exploratory finding should be interpreted cautiously. **Conclusions:** SGLT2 inhibitor use was independently associated with lower odds of NOAF after TAVI. These findings should be interpreted as observational and hypothesis-generating and require confirmation in prospective randomized studies.

## 1. Introduction

Aortic stenosis (AS) is characterized by chronic pressure overload, leading to left ventricular hypertrophy, left atrial enlargement, and progressive atrial remodeling [1,2]. These structural and electrophysiological changes predispose patients to atrial fibrillation (AF), particularly in advanced stages of the disease [1,2], and may contribute to atrial arrhythmogenesis during the periprocedural period following transcatheter aortic valve implantation (TAVI) [3].

TAVI has become an established treatment for severe symptomatic AS, and contemporary guideline recommendations now support its use across the entire surgical-risk spectrum. Following randomized trials demonstrating favorable outcomes across intermediate- and low-risk populations, current European and North American guidelines recommend TAVI as the preferred intervention in older patients suitable for a transfemoral approach and support its use in selected younger and lower-risk patients after multidisciplinary Heart Team evaluation [4]. Despite excellent procedural outcomes, heart failure (HF) remains a major source of morbidity after TAVI, reflecting persistent left ventricular hypertrophy and fibrosis, diastolic dysfunction, and microvascular dysfunction that may not fully resolve after relief of the valvular obstruction [5]. In real-world registries, the rate of HF hospitalization within one year of TAVI ranges from approximately 12% to 24%, and HF is consistently the leading cause of rehospitalization and is strongly associated with subsequent mortality in this population [6]. Consequently, therapeutic strategies capable of reducing both HF-related events and arrhythmic complications have become increasingly relevant in contemporary TAVI practice. Among these complications, new-onset atrial fibrillation (NOAF) is closely linked to this HF burden, both as a marker of advanced cardiac remodeling and as a precipitant of haemodynamic decompensation, and remains a frequent complication after the procedure, with a reported incidence ranging from 7% to 37% depending on monitoring strategy and patient characteristics [7,8,9]. NOAF after TAVI is associated with adverse clinical outcomes, including stroke, heart failure, and increased mortality [7,8,9,10].

Sodium–glucose cotransporter-2 (SGLT2) inhibitors have demonstrated broad cardiovascular benefits beyond glucose lowering, including favorable effects on ventricular and atrial remodeling, reduction in filling pressures, and attenuation of neurohormonal activation [11,12]. In addition to their well-established role in patients with diabetes mellitus and heart failure, emerging data suggest that SGLT2 inhibitors may be associated with a lower incidence of atrial fibrillation [13,14,15,16,17]. Accordingly, SGLT2 inhibitors are now guideline-recommended for heart failure across the full ejection-fraction spectrum and for chronic kidney disease, regardless of the presence of type 2 diabetes mellitus [18]. Although generally well tolerated, SGLT2 inhibitors may be associated with genital and urinary tract infections, volume depletion, euglycemic diabetic ketoacidosis, and, rarely, Fournier’s gangrene [19].

However, data on the potential antiarrhythmic effects of SGLT2 inhibitors specifically in the TAVI setting remain scarce. Therefore, the present study aimed to investigate the association between SGLT2 inhibitor use and the incidence of NOAF following TAVI.

## 2. Materials and Methods

### 2.1. Study Design and Population

This retrospective observational study included consecutive patients with severe aortic stenosis who underwent TAVI at our institution between January 2020 and December 2025. Severe aortic stenosis was defined according to current guideline recommendations [4]. All procedures were performed via the transfemoral approach.

Patients were eligible if they underwent transfemoral TAVI for severe symptomatic aortic stenosis and were in sinus rhythm at baseline. Baseline sinus rhythm was confirmed by 12-lead electrocardiography and review of available pre-procedural rhythm records. Patients with baseline atrial fibrillation, a documented history of paroxysmal atrial fibrillation or atrial flutter, non-transfemoral access, or incomplete clinical, laboratory, or follow-up data were excluded. To further exclude pre-existing atrial fibrillation, available previous electrocardiograms, hospitalization records, outpatient cardiology evaluations, discharge summaries, and medication histories were reviewed whenever accessible. In addition, chronic oral anticoagulant use was assessed, and the indication for anticoagulation was evaluated whenever available. Patients receiving oral anticoagulants because of documented atrial fibrillation or atrial flutter were excluded. These steps were undertaken to ensure accurate assessment of new-onset atrial fibrillation after TAVI. No imputation was performed for missing data. A total of 138 patients were excluded because of baseline atrial fibrillation or a documented history of paroxysmal atrial fibrillation. After application of the predefined inclusion and exclusion criteria, the final study population consisted of 573 patients in sinus rhythm at baseline.

The study protocol was approved by the local institutional ethics committee (approval number: 2026/04/1391) and was conducted in accordance with the principles of the Declaration of Helsinki. Informed consent was waived due to the retrospective nature of the study.

### 2.2. TAVI Procedure

All TAVI procedures were performed using standard techniques via the transfemoral route. Balloon aortic valvuloplasty before (pre-dilatation) and/or after (post-dilatation) valve implantation was performed at the discretion of the operator based on individual anatomical and procedural considerations. Both balloon-expandable and self-expanding transcatheter heart valves were used, with device type and valve size determined according to the operator’s discretion and anatomical suitability.

### 2.3. Data Collection

Clinical, laboratory, and medication data were obtained retrospectively from the institutional electronic medical records. Baseline demographic characteristics, comorbidities, laboratory parameters, and pharmacological treatments were recorded.

Use of sodium–glucose cotransporter-2 (SGLT2) inhibitors was defined as active treatment at the time of TAVI, regardless of the underlying indication. SGLT2 inhibitors could have been prescribed for diabetes mellitus, heart failure (including preserved or reduced ejection fraction), or other clinical indications. The most commonly used agents were empagliflozin and dapagliflozin. To be classified as an SGLT2 inhibitor user, patients were required to have been receiving therapy for at least 30 days prior to the TAVI procedure. Medication use was verified through review of medical records and medication lists. According to institutional practice, SGLT2 inhibitor therapy was routinely withheld approximately 72 h before the TAVI procedure and reinitiated after the procedure when clinically appropriate.

### 2.4. Definition of New-Onset Atrial Fibrillation

NOAF was defined as any episode of atrial fibrillation lasting ≥30 s occurring during the index hospitalization after TAVI in patients with no prior history of AF. AF episodes were identified using a standardized post-procedural monitoring protocol applied to all patients irrespective of SGLT2 inhibitor use. Continuous cardiac telemetry monitoring was performed throughout the intensive care unit stay following TAVI and during subsequent hospitalization according to institutional practice. In addition, serial 12-lead electrocardiograms were obtained daily and whenever symptoms suggestive of arrhythmia occurred. Patients were classified as having NOAF if AF was documented at any point between the procedure and hospital discharge.

### 2.5. Statistical Analysis

The normality of continuous variables was assessed using the Shapiro-Wilk test and visual inspection of histograms. Normally distributed variables are presented as mean ± standard deviation (SD), while non-normally distributed variables are expressed as median and interquartile range (IQR). Categorical variables are reported as counts and percentages (%). Baseline characteristics between groups were compared using Student’s *t*-test, Mann-Whitney U test, Chi-square test, or Fisher’s exact test, as appropriate for the data type and distribution.

To evaluate the association between SGLT2i use and post-procedural AF and to minimize selection bias and baseline clinical imbalances, a doubly robust estimation approach was utilized. A logistic regression model was constructed to estimate the probability (Propensity Score—PS) of receiving SGLT2i treatment. The propensity score model included age, sex, hypertension, coronary artery disease, diabetes mellitus, chronic kidney disease, COPD, heart failure, baseline LVEF, mitral and tricuspid regurgitation grades, systolic pulmonary artery pressure, TAPSE, hemoglobin, creatinine, and baseline medications (beta-blockers, ACE inhibitors/ARBs, ARNi, MRAs, insulin, furosemide, and statins).

Based on the estimated scores, Inverse Probability Weighting (IPW) was applied to calculate the Average Treatment Effect (ATE). To mitigate the influence of extreme weights, weights were stabilized, and 1% trimming was performed to exclude outliers. Balance between treatment groups was verified using Standardized Mean Differences (SMDs) and mirror density plots. To further enhance the reliability of the estimates, a multivariable logistic regression analysis was performed on the IPW-weighted cohort. This doubly robust model integrated the weights while adjusting for age, sex, hypertension, coronary artery disease, diabetes mellitus, chronic kidney disease, baseline LVEF, valve type, and baseline beta-blocker use. To assess potential multicollinearity between diabetes mellitus and SGLT2i use, variance inflation factors (VIF) were calculated for all covariates included in the multivariable model. VIF values below 5 were considered acceptable. To assess the consistency of the SGLT2i effect across different clinical phenotypes, a subgroup analysis was performed based on diabetes status (presence vs. absence). An interaction term was incorporated into the model to evaluate potential differences in treatment effect, and a p-interaction value was calculated. To further illustrate the association between SGLT2 inhibitor use and new-onset AF across clinical phenotypes, predicted probability curves were generated as a function of baseline LVEF, stratified by diabetes status and treatment group.

To assess the potential influence of unmeasured confounding on the primary association, an E-value was calculated. Because the outcome was not rare, the odds ratio was converted to an approximate risk ratio prior to E-value calculation. To further evaluate the robustness of the findings, a sensitivity analysis using 1:1 nearest-neighbor propensity score matching (without replacement, caliper 0.2 of the standard deviation of the logit of the propensity score) was also performed.

All statistical analyses were conducted using R Software (version 4.3.2; R Foundation for Statistical Computing, Vienna, Austria) utilizing the weightit, survey, rms, cobalt, ggeffects packages. Standard errors and *p*-values were calculated using robust sandwich variance estimators to account for the inverse probability weighting design. A two-sided *p*-value of <0.05 was considered statistically significant.

## 3. Results

### 3.1. Study Population and Baseline Characteristics

A total of 573 patients undergoing TAVI were included in the study, of whom 169 received SGLT2 inhibitors and 404 served as controls. Baseline characteristics are summarized in Table 1. Patients in the SGLT2i group were younger (median 77 vs. 79 years, *p* < 0.001) and had significantly higher rates of hypertension, coronary artery disease, diabetes mellitus, and COPD (all *p* < 0.05), with 82% of SGLT2i users having diabetes mellitus. SGLT2i users also had lower baseline LVEF and more frequent use of beta-blockers, statins, ARNI, MRA, insulin, and furosemide. Post-procedural AF occurred less frequently in the SGLT2i group compared with controls (11% vs. 19%, *p* = 0.041), whereas in-hospital mortality was similar between groups (6% vs. 8%, *p* = 0.448). Hospital length of stay was similar between the SGLT2 inhibitor and control groups (median 4.0 [IQR 3–7] vs. 4.0 [IQR 3–6] days, *p* = 0.72).

**Figure 1:**
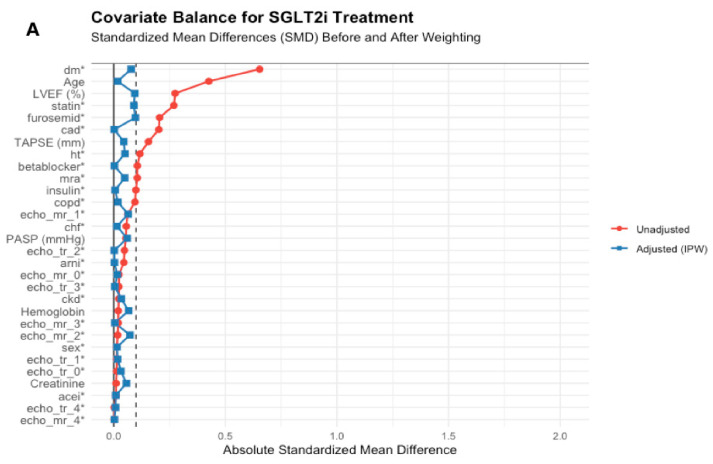

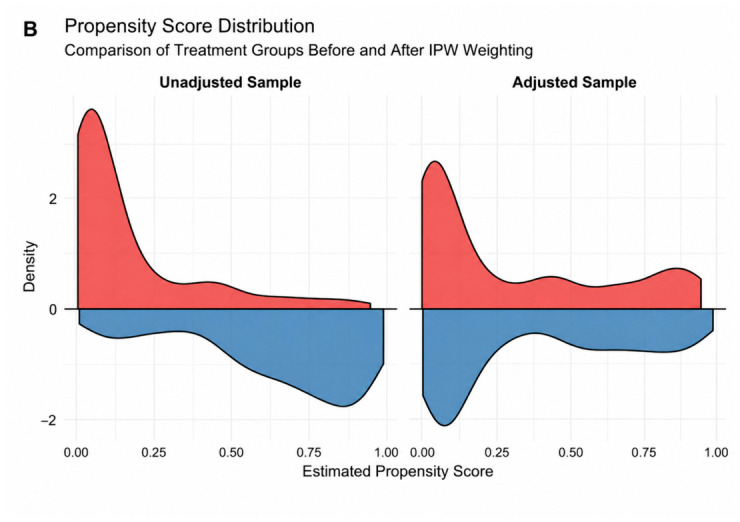
Covariate balance and propensity score distribution before and after inverse probability weighting (IPW) for SGLT2 inhibitor treatment. (**A**) Love plot showing absolute standardized mean differences (SMDs) for baseline covariates in the unweighted (red) and IPW-weighted (blue) cohorts. The dashed vertical line indicates the predefined balance threshold of 0.1. Several covariates exceeded this threshold before weighting but achieved SMDs < 0.1 after IPW adjustment, indicating improved covariate balance between treatment groups. Asterisks (*) denote categorical variables. (**B**) Mirrored density plots of estimated propensity scores according to treatment group before and after weighting (red: non-SGLT2i; blue: SGLT2i). Propensity score overlap improved substantially after weighting, suggesting adequate overlap between groups and supporting the positivity assumption. Abbreviations: ACEI, angiotensin-converting enzyme inhibitor; ARNI, angiotensin receptor–neprilysin inhibitor; CAD, coronary artery disease; CHF, congestive heart failure; CKD, chronic kidney disease; COPD, chronic obstructive pulmonary disease; DM, diabetes mellitus; IPW, inverse probability weighting; LVEF, left ventricular ejection fraction; MRA, mineralocorticoid receptor antagonist; NOAF, new-onset atrial fibrillation; PASP, pulmonary artery systolic pressure; SGLT2i, sodium-glucose cotransporter-2 inhibitor; SMD, standardized mean difference; TAPSE, tricuspid annular plane systolic excursion; TAVI, transcatheter aortic valve implantation.

Given the substantial baseline differences between groups, a doubly robust inverse probability weighting approach was applied. Stabilized inverse-probability weights were derived, and symmetric 1% trimming was applied to limit the influence of extreme weights. The stabilized weights ranged from 0.312 to 6.910 (median 0.726, mean 0.940). After IPW adjustment, covariate balance improved substantially, with all standardized mean differences reduced below the predefined threshold of 0.10 (maximum absolute SMD 0.097; Supplementary Table S1, Figure 1A). The effective sample size after weighting was 215.95 in the control group and 54.59 in the SGLT2 inhibitor group. Mirrored propensity-score density plots before and after weighting demonstrated adequate overlap and common support between treatment groups across the propensity-score distribution (Figure 1B).

### 3.2. Primary Outcome: Post-Procedural Atrial Fibrillation

**Figure 2:**
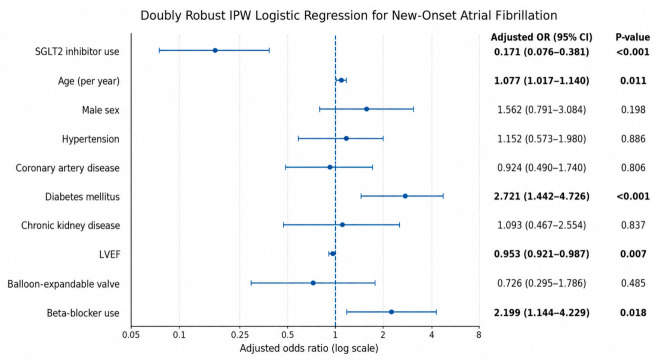
Forest plot of the doubly robust inverse probability-weighted (IPW) logistic regression model for new-onset atrial fibrillation (NOAF) following TAVI. Points represent adjusted odds ratios (aORs), and horizontal lines indicate 95% confidence intervals (CIs). The vertical dashed line at OR = 1 represents the null effect. The x-axis is displayed on a logarithmic scale. SGLT2 inhibitor use was independently associated with lower odds of NOAF, whereas older age and diabetes mellitus were associated with higher odds. Higher left ventricular ejection fraction (LVEF) was associated with lower odds of NOAF. Bold values indicate statistically significant associations (*p* < 0.05). Abbreviations: CI, confidence interval; IPW, inverse probability weighting; LVEF, left ventricular ejection fraction; NOAF, new-onset atrial fibrillation; OR, odds ratio; SGLT2i, sodium-glucose cotransporter-2 inhibitor; TAVI, transcatheter aortic valve implantation.

In the doubly robust weighted logistic regression analysis, SGLT2 inhibitor use was independently associated with lower odds of post-procedural AF (adjusted OR: 0.171, 95% CI: 0.076–0.381, *p* < 0.001). (Table 2, Figure 2). Older age (OR: 1.077 per year, 95% CI: 1.017–1.140, *p* = 0.011) and diabetes mellitus (OR: 2.721, 95% CI: 1.442–4.726, *p* < 0.001) were independently associated with higher odds of post-procedural AF, whereas higher baseline LVEF was associated with lower odds (OR: 0.953 per %, 95% CI: 0.921–0.987, *p* = 0.007). Beta-blocker use was also associated with increased odds of post-procedural AF (OR: 2.199, 95% CI: 1.144–4.229, *p* = 0.018). The E-value for the confidence limit closest to the null was 4.06. In a sensitivity analysis using 1:1 propensity score matching, the association remained consistent (adjusted OR 0.179, 95% CI 0.077–0.414, *p* < 0.001).

In the propensity score-weighted cohort, the adjusted absolute risk of post-procedural AF was 29.1% in the control group versus 9.3% in the SGLT2i group, corresponding to an adjusted risk difference of −19.7% (95% CI: −28.9% to −10.5%, *p* < 0.001). With respect to the safety endpoints, the adjusted risk difference was −13.0% (95% CI: −29.0% to 3.0%, *p* = 0.111) for postoperative AKI and −4.3% (95% CI: −10.2% to 1.6%, *p* = 0.156) for in-hospital mortality; neither difference reached statistical significance. The adjusted odds ratios, absolute risks, and risk differences for all outcomes are summarized in Table 3.

Subgroup analysis demonstrated a significant interaction according to diabetes status (P-interaction = 0.040). In the diabetic subgroup, new-onset AF occurred in 36/64 (56.3%) of controls versus 17/139 (12.2%) of SGLT2 inhibitor users, corresponding to a significantly lower risk of post-procedural AF among patients receiving SGLT2 inhibitors (adjusted OR 0.078, 95% CI 0.028–0.217, *p* < 0.001). In the non-diabetic subgroup, new-onset AF occurred in 39/340 (11.5%) of controls versus 2/30 (6.7%) of SGLT2 inhibitor users (adjusted OR 0.417, 95% CI 0.147–1.183, *p* = 0.096) (Table 4) The corresponding subgroup forest plot is presented in Supplementary Figure S1.

**Figure 3:**
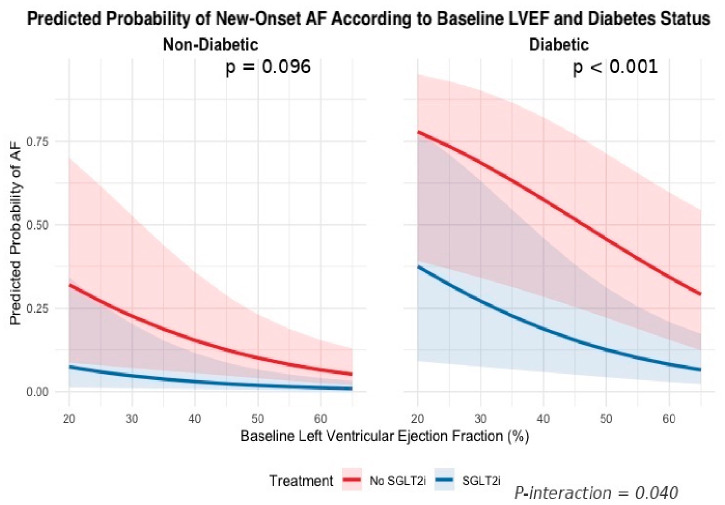
Predicted probability of new-onset atrial fibrillation (NOAF) following TAVI according to baseline left ventricular ejection fraction (LVEF), stratified by diabetes status and SGLT2 inhibitor treatment. Solid lines represent model-predicted probabilities derived from the doubly robust inverse probability-weighted logistic regression model, and shaded areas indicate 95% confidence bands. Red denotes patients not receiving SGLT2i, whereas blue denotes SGLT2i-treated patients. The predicted probability of NOAF decreased with increasing LVEF in both subgroups. The association between SGLT2 inhibitor use and lower predicted NOAF risk appeared more pronounced among diabetic patients, consistent with the statistically significant treatment-by-diabetes interaction (P-interaction = 0.040). Abbreviations: LVEF, left ventricular ejection fraction; NOAF, new-onset atrial fibrillation; SGLT2i, sodium-glucose cotransporter-2 inhibitor; TAVI, transcatheter aortic valve implantation.

Among diabetic patients, SGLT2 inhibitor use was associated with a markedly lower predicted probability of post-procedural AF across the observed range of baseline LVEF. In contrast, although a numerically lower AF probability was also observed in non-diabetic patients receiving SGLT2 inhibitors, this association did not reach statistical significance. In both subgroups, lower baseline LVEF was associated with a progressively higher predicted probability of new-onset AF, with the highest predicted risk observed among diabetic patients not receiving SGLT2 inhibitors. The corresponding predicted probability curves of post-procedural AF across baseline LVEF, stratified by diabetes status and treatment group, are presented in Figure 3.

Although the interaction test reached statistical significance, these subgroup findings should be interpreted cautiously given the limited number of non-diabetic patients receiving SGLT2 inhibitors (*n* = 30) and the low event count within this subgroup (*n* = 2).

## 4. Discussion

In this retrospective observational study, of patients undergoing TAVI, pre-procedural SGLT2 inhibitor use was independently associated with lower odds of new-onset atrial fibrillation following TAVI. This association persisted after adjustment for baseline confounders using a doubly robust inverse probability weighting approach. Beyond the primary analysis, subgroup analyses suggested that the association may be stronger among patients with diabetes mellitus; however, these exploratory findings should be interpreted cautiously given the limited number of non-diabetic patients receiving SGLT2 inhibitors. Taken together, these findings support a possible association between pre-procedural SGLT2 inhibitor use and lower post-procedural atrial fibrillation risk in patients undergoing TAVI.

The present study has several notable strengths. First, it addresses a clinically relevant question regarding the association between SGLT2 inhibitor use and new-onset atrial fibrillation following TAVI, a setting in which available data remain limited. Second, we applied a doubly robust inverse probability weighting approach to reduce baseline differences between treatment groups and improve the validity of the observed associations. Finally, the study reflects contemporary real-world clinical practice in a well-characterized TAVI population.

New-onset atrial fibrillation remains one of the most frequent complications following TAVI, with reported incidence rates ranging between 7% and 37% depending on patient characteristics, rhythm monitoring strategies, and AF definitions used across studies [7,8,9,10]. In the present cohort, post-procedural AF occurred in approximately 16% of patients, consistent with previous TAVI registries and observational studies. However, the clinical significance of NOAF extends well beyond its incidence. New-onset atrial fibrillation following TAVI has consistently been associated with increased risks of thromboembolic events, particularly stroke, as well as higher short-term mortality and prolonged hospitalization [7,8,9,10]. Given these adverse clinical consequences, the management of NOAF after TAVI has become an increasingly important aspect of post-procedural care.

Importantly, because no dedicated guideline recommendations exist for atrial fibrillation arising specifically after TAVI, its management remains challenging and is largely extrapolated from general atrial fibrillation guidelines [20]. Rate- or rhythm-control strategies are typically selected according to symptoms and hemodynamic status; however, therapeutic decision-making is often complicated by advanced age, frailty, multiple comorbidities, and the frequent occurrence of conduction disturbances requiring permanent pacemaker implantation in this population. Decisions regarding oral anticoagulation are similarly challenging, as the thromboembolic benefit must be balanced against bleeding risk in elderly patients who are frequently receiving concomitant antiplatelet therapy after the procedure [8,21]. Furthermore, a substantial proportion of post-procedural AF may be transient and peri-procedural in nature, leaving uncertainty regarding the optimal duration of anticoagulation and the role of prolonged rhythm monitoring after discharge. Consequently, the absence of TAVI-specific management pathways may contribute to variability in clinical practice. These challenges highlight the potential value of preventive strategies aimed at reducing the occurrence of NOAF, rather than relying solely on its treatment once established.

SGLT2 inhibitors have demonstrated broad cardiovascular benefits extending beyond glycemic control, including favorable effects on ventricular loading conditions, myocardial metabolism, inflammation, oxidative stress, and neurohormonal activation [11,12]. In addition to their established role in patients with diabetes mellitus and heart failure, accumulating evidence suggests that SGLT2 inhibitors may also be associated with lower rates of atrial fibrillation across different clinical settings [15,16,17,22]. Several experimental and clinical studies have proposed multiple mechanisms underlying this potential antiarrhythmic effect, including attenuation of atrial fibrosis and remodeling, reduction in left atrial pressure and volume overload, improvement in autonomic balance, and suppression of systemic inflammation and oxidative stress [11,12,13,14,23]. Moreover, prior studies conducted in patients with diabetes mellitus, heart failure, and acute coronary syndromes have reported lower rates of incident or recurrent AF among patients receiving SGLT2 inhibitors [16,17,24,25,26,27,28,29]. Consistent with these observations, a recent device-based study reported a substantial reduction in both atrial and ventricular arrhythmic events following SGLT2 inhibitor initiation in patients with heart failure and cardiac implantable electronic devices, providing further support for a potential antiarrhythmic effect of this drug class [30]. In the specific context of TAVI, the DapaTAVI trial recently demonstrated that dapagliflozin significantly reduced the composite of all-cause mortality and worsening heart failure at one year, further supporting a disease-modifying role of SGLT2 inhibitors in this population [31]. Given that patients undergoing TAVI are particularly susceptible to atrial pressure overload, inflammatory activation, and peri-procedural hemodynamic stress, these pleiotropic effects may be especially relevant in this setting [2,3]. However, because the present study did not directly assess atrial remodeling, left atrial or filling pressures, inflammatory or oxidative-stress markers, autonomic function, or volume status, these mechanisms remain hypothetical and cannot be confirmed by our data.

Beyond SGLT2 inhibitors, it is plausible that optimization of guideline-directed heart failure therapy may also contribute to a lower arrhythmic burden in patients undergoing TAVI. Several mechanisms proposed to explain the potential antiarrhythmic effects of SGLT2 inhibitors, including reduction of filling pressures, attenuation of neurohormonal activation, and reverse cardiac remodeling, may also be achieved through other established heart failure therapies, including pharmacological treatment and cardiac rehabilitation [32]. Therefore, although SGLT2 inhibitors have been hypothesized to exert specific antiarrhythmic effects, the association observed in the present study could also partly reflect the broader benefits of comprehensive heart failure management. Future studies should further investigate the relative contribution of different heart failure therapies to the risk of NOAF following TAVI.

In our cohort, SGLT2 inhibitor use was independently associated with lower odds of NOAF. This association is biologically plausible given the known pleiotropic cardiovascular effects of this drug class; however, the observational nature of the present study does not permit causal inference. Subgroup analyses suggested a potentially stronger association among patients with diabetes mellitus, with a statistically significant interaction according to diabetic status. However, these findings should be interpreted cautiously because the number of non-diabetic patients receiving SGLT2 inhibitors was relatively limited, which may have reduced statistical power in this subgroup. In contrast, although the direction of the association remained consistent with lower odds of NOAF in non-diabetic patients, it did not reach statistical significance. This differential pattern warrants careful interpretation. Given that 82% of patients in the SGLT2i group had diabetes mellitus, the observed association in the overall cohort was likely driven predominantly by the diabetic subgroup, and the apparent attenuation of association in non-diabetic patients may partly reflect the substantially smaller number of non-diabetic patients receiving SGLT2 inhibitors rather than a true absence of effect. Indeed, with the recent expansion of SGLT2 inhibitor indications to include heart failure regardless of glycemic status, future studies enrolling larger proportions of non-diabetic TAVI patients receiving SGLT2 inhibitors will be important to determine whether this association extends beyond the diabetic substrate.

Diabetes mellitus is a well-established risk factor for atrial fibrillation and has been consistently associated with adverse outcomes following TAVI [33,34]. Multiple pathophysiological mechanisms may contribute to the increased susceptibility to AF in diabetic patients, including chronic systemic inflammation, oxidative stress, autonomic dysfunction, impaired diastolic function, atrial fibrosis, and structural atrial remodeling [23,34]. In line with these observations, diabetes mellitus emerged as one of the strongest independent predictors of post-procedural AF in our cohort. Although the subgroup estimates should be interpreted with caution given the retrospective nature of the study, the magnitude of the observed association further supports the concept that diabetic patients undergoing TAVI may represent a particularly arrhythmia-prone subgroup. In this context, it is conceivable that the anti-inflammatory, antifibrotic, and hemodynamic properties of SGLT2 inhibitors may be particularly relevant in diabetic patients, potentially corresponding to the more pronounced association observed in this subgroup [11,12,14,16]. Interestingly, beta-blocker use was also associated with a higher risk of post-procedural AF in the adjusted analysis. However, this finding likely reflects residual confounding by indication and greater baseline cardiovascular disease burden, as beta-blockers are more commonly prescribed in patients with heart failure or a more advanced arrhythmogenic substrate rather than representing a direct proarrhythmic effect. This association should therefore be interpreted with caution and not as evidence of a causal proarrhythmic role for beta-blockers in this setting.

The findings of the present study may carry important clinical implications. If confirmed in larger prospective studies, SGLT2 inhibitor use may identify a subgroup of with lower post-procedural AF risk in patients undergoing TAVI. Given that SGLT2 inhibitors are already indicated in a substantial proportion of TAVI candidates, particularly those with diabetes mellitus, heart failure, or chronic kidney disease, the association observed in the present study, if confirmed, might be relevant without additional pharmacological burden. Furthermore, with the expanding indications of SGLT2 inhibitors beyond glycemic control, future studies will be important to determine whether the observed association extends to TAVI patients regardless of diabetic status.

Beyond the relative effect estimates, expressing the association on an absolute scale provides additional clinical context. In the propensity score-weighted cohort, the adjusted absolute risk of post-procedural AF was nearly 20 percentage points lower among patients receiving SGLT2 inhibitors. Although causal inference cannot be established from the present observational study, an effect of this magnitude, if confirmed in prospective investigations, could be clinically relevant in the peri-procedural TAVI setting. With respect to safety, no statistically significant differences were observed in postoperative AKI or in-hospital mortality between the two groups. While these analyses were not powered for safety outcomes and should be considered exploratory, no apparent early safety signal associated with SGLT2 inhibitor use was identified.

However, the present study was not designed to establish causality, and decisions regarding initiation or continuation of SGLT2 inhibitor therapy in the peri-procedural setting should continue to be guided by current clinical guidelines and individualized patient assessment until prospective data become available.

## 5. Limitations

Several limitations of the present study should be acknowledged. First, the retrospective single-center design inherently limits generalizability and precludes causal inference. Although a doubly robust inverse probability weighting approach, E-value analysis, and propensity score–matched sensitivity analyses were applied, residual confounding and confounding by indication cannot be completely excluded. Second, rhythm monitoring was limited to the index hospitalization. Systematic ambulatory monitoring after discharge was unavailable, and prolonged pre-procedural rhythm surveillance was not uniformly performed. Consequently, both previously undiagnosed paroxysmal AF and asymptomatic AF episodes occurring after discharge may have been missed, potentially resulting in outcome misclassification and underestimation of the true AF burden. Third, detailed information regarding the duration of SGLT2 inhibitor therapy, specific agents, dosing regimens, and long-term treatment adherence was not systematically available. Therefore, agent-specific, dose-dependent, and exposure-duration effects could not be evaluated. Fourth, several clinically relevant outcomes, including stroke, bleeding complications, permanent pacemaker implantation, and other procedure-related adverse events, were not systematically captured within the institutional registry and therefore could not be analyzed. As a result, a more comprehensive assessment of the overall clinical impact and safety profile associated with SGLT2 inhibitor use following TAVI was not possible. Fifth, several procedural and peri-procedural variables known to influence post-procedural AF risk, including pre-dilatation, post-dilatation, rapid ventricular pacing, anesthesia type, conduction disturbances, electrolyte abnormalities, transfusion requirements, and procedural complications, were not systematically recorded and therefore could not be incorporated into the analyses. Finally, the relatively small sample size, particularly within certain subgroups, may have limited statistical power and contributed to imprecision in subgroup-specific estimates.

## 6. Conclusions

SGLT2 inhibitor use was independently associated with lower odds of new-onset atrial fibrillation following TAVI. This association appeared more pronounced among patients with diabetes mellitus, although residual confounding cannot be excluded. These findings support the hypothesis that SGLT2 inhibitors may be associated with antiarrhythmic effects in patients undergoing TAVI and warrant confirmation in prospective randomized studies.

## Figures and Tables

**Table 1:** Baseline Clinical, Echocardiographic, Laboratory, and Procedural Characteristics According to SGLT2i Use.

Variable	Control Group (*n* = 404)	SGLT2i Group (*n* = 169)	*p*-Value
Demographics			
Age, years	79.0 [75.0–83.0]	77.0 [73.0–81.0]	**<0.001**
Male sex, *n* (%)	196 (49%)	81 (48%)	0.971
Comorbidities			
Hypertension, *n* (%)	323 (80%)	155 (92%)	**<0.001**
Coronary artery disease, *n* (%)	188 (47%)	109 (64%)	**<0.001**
Diabetes mellitus, *n* (%)	64 (16%)	139 (82%)	**<0.001**
Heart failure, *n* (%)	67 (17%)	36 (21%)	0.222
COPD, *n* (%)	44 (11%)	34 (20%)	**0.005**
Chronic kidney disease, *n* (%)	58 (14%)	30 (18%)	0.368
Cerebrovascular accident, *n* (%)	22 (5%)	8 (5%)	0.886
Pre-procedural Echocardiography			
LVEF, %	65.0 [55.0–65.0]	60.0 [45.0–65.0]	**0.031**
LVEDD, cm	4.6 [4.3–5.1]	4.8 [4.5–5.2]	**0.002**
LVESD, cm	2.9 [2.5–3.4]	3.1 [2.7–3.8]	**0.001**
Mitral Regurgitation (≥Grade 2), *n* (%)	173 (43%)	83 (49%)	0.197
Aortic Regurgitation (≥Grade 2), *n* (%)	181 (45%)	87 (51%)	**0.029**
sPAP, mmHg	35.0 [30.0–50.0]	35.0 [25.0–50.0]	0.323
Mean Aortic Gradient, mmHg	44.0 [40.0–52.0]	43.0 [40.0–49.0]	0.095
TAPSE, mm	2.0 [1.8–2.2]	2.0 [1.8–2.1]	0.163
Low-Flow Low-Gradient (LFLG), *n* (%)	44 (11%)	29 (17%)	0.057
Laboratory Parameters			
Hemoglobin, g/dL	11.7 ± 1.9	11.7 ± 2.0	0.998
Creatinine, mg/dL	1.0 [0.8–1.2]	0.9 [0.8–1.3]	0.474
Albumin, g/dL	4.0 [3.8–4.2]	4.0 [3.8–4.2]	0.286
Pro-BNP, pg/mL	520 [140–1197]	583 [137–1535]	0.957
CRP, mg/L	5.0 [2.0–13.0]	4.4 [1.9–11.6]	0.547
WBC, ×10^3^/µL	7.3 [6.0–9.0]	7.4 [5.9–9.0]	0.680
Medications			
Beta-blockers, *n* (%)	236 (58%)	115 (68%)	**0.039**
ACEi/ARBs, *n* (%)	302 (75%)	126 (75%)	1.000
Statins, *n* (%)	131 (32%)	96 (57%)	**<0.001**
ARNI, *n* (%)	4 (1%)	10 (6%)	**0.001**
MRA, *n* (%)	30 (7%)	30 (18%)	**<0.001**
Insulin, *n* (%)	24 (6%)	26 (15%)	**<0.001**
Furosemide, *n* (%)	99 (25%)	77 (46%)	**<0.001**
Procedural and Post-op			
Balloon-expandable valve, *n* (%)	82 (20%)	36 (21%)	0.896
Agatston Score (CT)	3227 [2199–4494]	2724 [2002–4047]	**0.024**
New-onset atrial fibrillation (NOAF), *n* (%)	75 (19%)	19 (11%)	**0.041**
In-hospital mortality, *n* (%)	33 (8%)	10 (6%)	0.448

Data are presented as mean ± standard deviation, median [interquartile range], or number (%), as appropriate. Bold values indicate statistical significance (*p* < 0.05) Abbreviations: SGLT2i, sodium-glucose cotransporter-2 inhibitor; COPD, chronic obstructive pulmonary disease; LVEF, left ventricular ejection fraction; LVEDD, left ventricular end-diastolic diameter; LVESD, left ventricular end-systolic diameter; sPAP, systolic pulmonary artery pressure; TAPSE, tricuspid annular plane systolic excursion; LFLG, low-flow low-gradient; CRP, C-reactive protein; WBC, white blood cell count; ACEi, angiotensin-converting enzyme inhibitor; ARB, angiotensin receptor blocker; ARNI, angiotensin receptor-neprilysin inhibitor; MRA, mineralocorticoid receptor antagonist; CT, computed tomography.

**Table 2:** Doubly Robust Inverse Probability Weighted Logistic Regression Analysis for New-Onset Atrial Fibrillation.

Variable	Adjusted Odds Ratio	95% CI	*p*-Value
SGLT2 inhibitor use	0.171	[0.076, 0.381]	<0.001
Age (per year)	1.077	[1.017, 1.140]	0.011
Male sex	1.562	[0.791, 3.084]	0.198
Hypertension	1.152	[0.573, 1.980]	0.886
Coronary artery disease	0.924	[0.490, 1.740]	0.806
Diabetes mellitus	2.721	[1.442, 4.726]	<0.001
Chronic kidney disease	1.093	[0.467, 2.554]	0.837
LVEF	0.953	[0.921, 0.987]	0.007
Balloon-expandable valve	0.726	[0.295, 1.786]	0.485
Beta-blocker use	2.199	[1.144, 4.229]	0.018

Adjusted odds ratios were obtained from a doubly robust inverse probability weighted logistic regression model incorporating clinically relevant covariates. Abbreviations: CI, confidence interval; LVEF, left ventricular ejection fraction.

**Table 3:** Adjusted clinical outcomes in the propensity score-weighted cohort.

Outcome	Control Group Rate	SGLT2i Group Rate	Adjusted Odds Ratio (95% CI)	Adjusted Risk Difference (95% CI)	*p*-Value
Postoperative AF	29.1%	9.3%	0.171 [0.076, 0.381]	−19.7% [−28.9%, −10.5%]	<0.001
Postoperative AKI	16.6%	3.6%	0.854 [0.412, 1.768]	−13.0% [−29.0%, 3.0%]	0.111
In-Hospital Mortality	7.3%	3.0%	0.702 [0.285, 1.724]	−4.3% [−10.2%, 1.6%]	0.156

Note: Estimates were derived from the propensity score-weighted outcome models. The adjusted absolute risks for the control and SGLT2i groups correspond to the model intercept and the combined intercept plus treatment coefficient, respectively. The adjusted risk difference (RD) represents the absolute difference in risk associated with SGLT2i use, with a negative value favoring SGLT2i. Confidence intervals were estimated from the weighted models, and *p*-values < 0.05 were considered statistically significant. Abbrevations: CI, confidence interval; SGLT2i, sodium-glucose cotransporter-2 inhibitor; AF, atrial fibrillation; AKI, acute kidney injury.

**Table 4:** Subgroup and Interaction Analyses of the Association Between SGLT2 Inhibitor Use and Post-Procedural New-Onset Atrial Fibrillation.

Subgroup	Adjusted OR	95% CI	*p*-Value	P-Interaction
Overall Population	0.171	[0.076, 0.381]	<0.001	
Diabetes Mellitus				0.040
Yes	0.078	[0.028, 0.217]	<0.001	
No	0.417	[0.147, 1.183]	0.096	
Baseline LVEF				0.086
<40%	0.252	[0.051, 1.236]	0.088	
≥40%	0.085	[0.031, 0.233]	<0.001	

Odds ratios were derived from inverse probability weighted logistic regression models. P interaction values represent tests for effect modification across subgroup categories. An OR < 1 indicates a lower odds of post-procedural new-onset atrial fibrillation associated with SGLT2 inhibitor use. Abbreviations: OR, odds ratio; CI, confidence interval; LVEF, left ventricular ejection fraction.

## Data Availability

The datasets used and/or analyzed during the current study are not publicly available due to institutional and ethical restrictions but are available from the corresponding author upon reasonable request and with additional institutional permission where required.

## Supplementary Materials

The following supporting information can be downloaded at: https://www.mdpi.com/article/10.3390/jcm15124812/s1, Figure S1: Subgroup analysis of the association between SGLT2 inhibitor use and new-onset atrial fibrillation after TAVI. Forest plot showing adjusted odds ratios (ORs) and 95% confidence intervals (CIs) for the association between SGLT2 inhibitor (SGLT2i) use and new-onset atrial fibrillation (NOAF) following transcatheter aortic valve implantation (TAVI) in the overall study population and across predefined subgroups. Estimates were derived from multivariable-adjusted models within each subgroup. Interaction *p*-values are presented to assess heterogeneity of treatment effect between subgroup categories. The association between SGLT2i use and lower NOAF risk appeared stronger among patients with diabetes mellitus (*p* for interaction = 0.040), whereas no statistically significant interaction was observed according to baseline left ventricular ejection fraction (LVEF) (*p* for interaction = 0.086). The vertical dashed line indicates an OR of 1.0 (no association); Table S1: Covariate Balance Before and After Inverse Probability Weighting.
