# Supplementary material for: Association Between SGLT2 Inhibitor Use and New-Onset Atrial Fibrillation Following Transcatheter Aortic Valve Implantation: A Doubly Robust Inverse Probability Weighted Analysis
Source: J Clin Med. 2026 Jun 21;15(12):4812. doi: 10.3390/jcm15124812 (PMC13302004; doi:10.3390/jcm15124812)
Supplement: Supplementary file 1 [file jcm-15-04812-s001.zip › jcm-4365786-supplementary.pdf]

Table S1. Covariate Balance Before and After Inverse Probability Weighting.

Baseline Covariates	Absolute SMD (Unadjusted)	Absolute SMD (Adjusted/IPW)
Age	0.426	0.018
Sex	0.014	0.015
Hypertension	0.117	0.050
Coronary Artery Disease	0.202	0.002
Diabetes Mellitus	0.653	0.078
Heart Failure	0.056	0.014
COPD	0.095	0.018
Chronic Kidney Disease	0.022	0.034
LVEF (%)	0.275	0.094
Mitral Regurgitation (Grade 0)	0.023	0.016
Mitral Regurgitation (Grade 1)	0.063	0.065
Mitral Regurgitation (Grade 2)	0.018	0.073
Mitral Regurgitation (Grade 3)	0.020	0.005
Mitral Regurgitation (Grade 4)	0.002	0.003
Tricuspid Regurgitation (Grade 0)	0.013	0.032
Tricuspid Regurgitation (Grade 1)	0.014	0.017
Tricuspid Regurgitation (Grade 2)	0.048	0.002
Tricuspid Regurgitation (Grade 3)	0.023	0.005
Tricuspid Regurgitation (Grade 4)	0.002	0.008
sPAP (mmHg)	0.055	0.060
TAPSE (mm)	0.155	0.045
Hemoglobin	0.020	0.067
Creatinine	0.010	0.057
Beta-blocker	0.106	0.003
ACEi/ARB	0.010	0.009
ARNI	0.045	0.003
MRA	0.105	0.049
Furosemide	0.205	0.097
Statin	0.270	0.090
Insulin	0.099	0.006

SMD values are presented as absolute values. Values <0.10 after weighting indicate adequate covariate balance. Abbreviations: SMD, standardized mean difference; IPW, inverse probability weighting; COPD, chronic obstructive pulmonary disease; LVEF, left ventricular ejection fraction; sPAP, systolic pulmonary artery pressure; TAPSE, tricuspid annular plane systolic excursion; ACEi/ARB, angiotensin-converting enzyme inhibitor/angiotensin receptor blocker; ARNI, angiotensin receptor-neprilysin inhibitor; MRA, mineralocorticoid receptor antagonist.

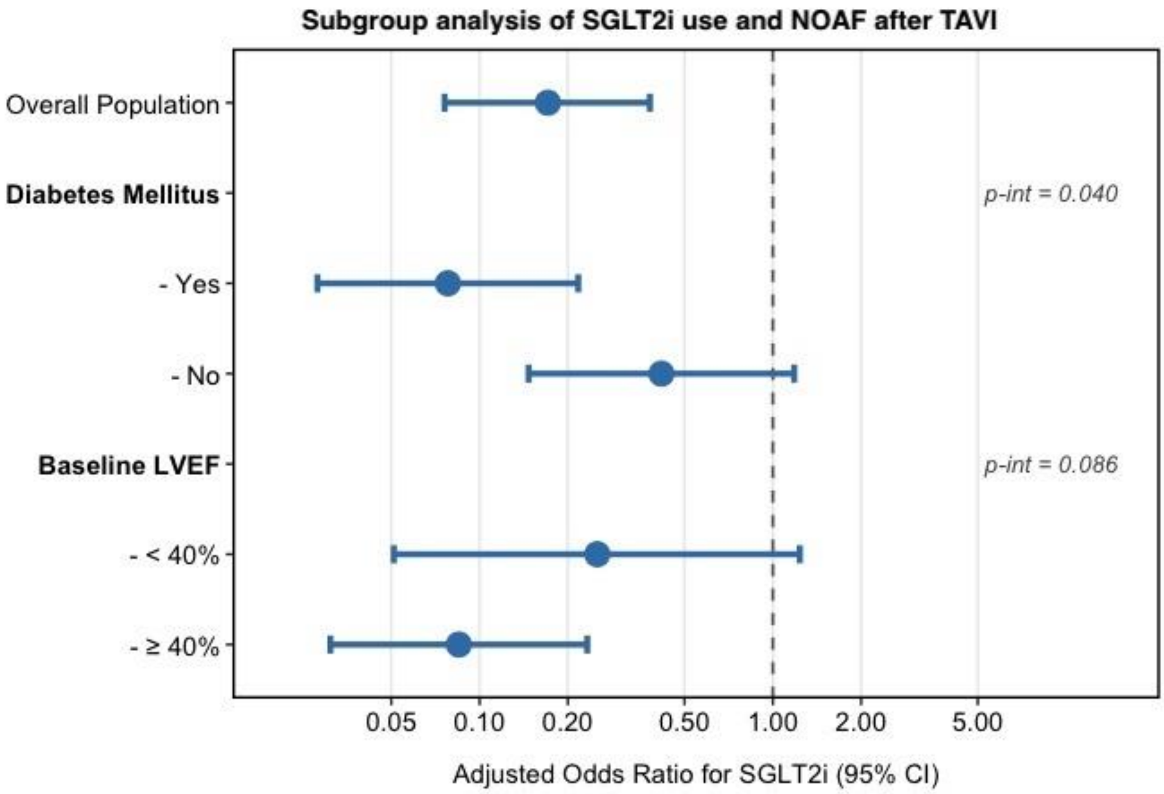

Figure S1. Subgroup analysis of the association between SGLT2 inhibitor use and new-onset atrial fibrillation after TAVI. Forest plot showing adjusted odds ratios (ORs) and 95% confidence intervals (CIs) for the association between SGLT2 inhibitor (SGLT2i) use and new-onset atrial fibrillation (NOAF) following transcatheter aortic valve implantation (TAVI) in the overall study population and across predefined subgroups. Estimates were derived from multivariable-adjusted models within each subgroup. Interaction p-values are presented to assess heterogeneity of treatment effect between subgroup categories. The association between SGLT2i use and lower NOAF risk appeared stronger among patients with diabetes mellitus (p for interaction = 0.040), whereas no statistically significant interaction was observed according to baseline left ventricular ejection fraction (LVEF) (p for interaction = 0.086). The vertical dashed line indicates an OR of 1.0 (no association).
